# Doxorubicin-induced DNA Damage Causes Extensive Ubiquitination of Ribosomal Proteins Associated with a Decrease in Protein Translation*[Fn FN1]

**DOI:** 10.1074/mcp.RA118.000652

**Published:** 2018-02-08

**Authors:** Vincentius A. Halim, Iraia García-Santisteban, Daniel O. Warmerdam, Bram van den Broek, Albert J. R. Heck, Shabaz Mohammed, René H. Medema

**Affiliations:** From the ‡Biomolecular Mass Spectrometry and Proteomics Group, Bijvoet Center for Biomolecular Research and Utrecht Institute for Pharmaceutical Sciences, Utrecht University, 3584 CH Utrecht, The Netherlands;; §Netherlands Proteomics Centre, 3584 CH Utrecht, The Netherlands;; ¶Division of Cell Biology and Cancer Genomics Center, Netherlands Cancer Institute, 1066 CX Amsterdam, The Netherlands;; ‖Department of Genetics, Physical Anthropology and Animal Physiology, University of the Basque Country (UPV/EHU), Leioa, Spain;; **European Research Institute for the Biology of Ageing, University Medical Center Groningen, 9713 AV Groningen, The Netherlands;; ‡‡Department of Biochemistry,; §§Chemistry Research Laboratory, Department of Chemistry, University of Oxford, OX13TA Oxford, United Kingdom

**Keywords:** Cell cycle*, Mass Spectrometry, Pathway Analysis, Ribosomes*, Ubiquitin

## Abstract

Protein posttranslational modifications (PTMs) play a central role in the DNA damage response. In particular, protein phosphorylation and ubiquitination have been shown to be essential in the signaling cascade that coordinates break repair with cell cycle progression. Here, we performed whole-cell quantitative proteomics to identify global changes in protein ubiquitination that are induced by DNA double-strand breaks. In total, we quantified more than 9,400 ubiquitin sites and found that the relative abundance of ∼10% of these sites was altered in response to DNA double-strand breaks. Interestingly, a large proportion of ribosomal proteins, including those from the 40S as well as the 60S subunit, were ubiquitinated in response to DNA damage. In parallel, we discovered that DNA damage leads to the inhibition of ribosome function. Taken together, these data uncover the ribosome as a major target of the DNA damage response.

The genome of a cell is frequently damaged by insults generated by internal (reactive cell metabolites) and external (irradiation, UV) sources (1–3). This causes a threat to the stability of the genome and can contribute to cancer development. To protect themselves against this potential threat, cells are equipped with powerful surveillance mechanisms that detect and repair the damage before it is propagated to subsequent daughter cells. This response is collectively referred to as the DNA damage checkpoint or the DNA damage response (1–3). Notwithstanding these harmful effects of DNA damage, several DNA damaging agents are widely applied to treat cancer, as they can cause a nonreversible checkpoint arrest or trigger cell death, thus curbing the rapid proliferation in cancer cells. For example, doxorubicin, which induces DNA double-strand breaks, is a potent anticancer drug that is commonly used in the clinic.

Execution of the DNA damage response requires damage detection, and initiates a signaling cascade that halts further progression through the cell cycle, while promoting repair. This signaling cascade is mainly driven by posttranslational modifications (4, 5). The phosphatidylinositol 3-kinase-related kinases ataxia-telangiectasia mutated (ATM) and ataxia- and Rad3- related (ATR) play a central role in the initiation of DNA damage response signaling. ATM and ATR are recruited to sites of DNA damage and subsequently phosphorylate over 700 substrates (6). Important downstream targets of ATM and ATR are Chk2 and Chk1 kinases, respectively (3). Phosphorylation of these effector kinases results in their activation, causing a subsequent wave of protein phosphorylations that are essential for the function of the DNA damage checkpoint and promotes cell cycle arrest (3, 6, 7).

A recent proteomics study showed that ubiquitination events following DNA damage are as common as phosphorylation (8). It is well established that in response to a double-strand break, ubiquitin-mediated signaling is initiated by the ubiquitin ligases RNF8 and RNF168 (9–13). Whereas phosphorylation events spread rapidly throughout the nucleus after damage, ubiquitination seems mostly limited to the proximity of the break site and more controlled (10, 12). Protein ubiquitination is essential for the buildup of the checkpoint and plays an important role in various repair pathways. Excessive protein ubiquitination can be detrimental for the maintenance of the DNA damage checkpoint and DNA repair pathway choice (14–16).

There are several reports showing that individual ribosomal proteins play a role in the DNA damage response, most notably in the activation of p53 (17, 18), but so far there are few reports that connect the DNA damage response with ribosomal function. DNA damage is known to affect mRNA translation by disruption of cap initiation complexes that are required for the recruitment of mRNAs to ribosomes (19, 20). In addition, DNA damaging agents have been shown to affect signaling through the mTOR pathway and, in consequence, also protein translation. In fact, the effects of irradiation on translation are much more pronounced than on transcription (21). These changes in translation are at least in part mediated through altered recruitment of mRNAs to polysomes (21), but how this is established is not known.

The eukaryotic 80S ribosome consists of four ribosomal RNAs (rRNAs) and 80 ribosomal proteins (RPs) (22–24). After their transcription in the nucleoli, rRNAs associate with RPS and RPL proteins in the nucleus, forming the small (40S) and large (60S) ribosomal subunits, respectively (22–24). The small and large subunits will be assembled together in the cytoplasm to make a mature ribosome (or monosome) (22–24). Importantly, several ribosomes can simultaneously translate a single mRNA molecule to synthesize the same protein, forming the so-called polysomes (22, 23).

In this study, we investigate how specific ubiquitination events change after DNA damage and identify the ribosome as a target of the DNA damage checkpoint.

## MATERIALS AND METHODS

### 

#### 

##### Cell Culture, Transfection, and Drugs

**Fig. 1.**
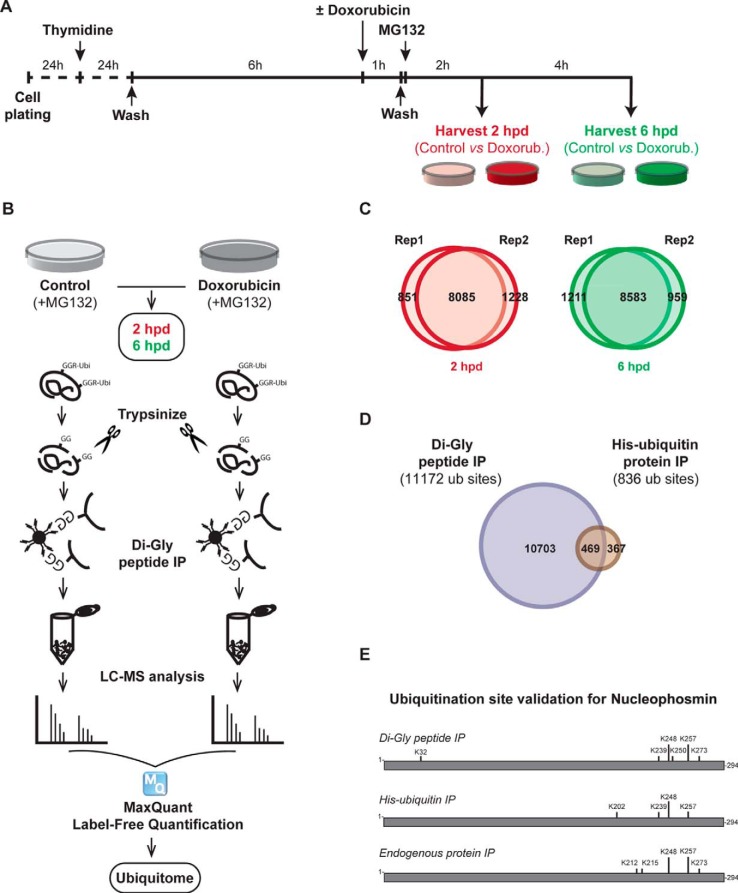
**Experimental setting, proteomics analysis, and validation.** (*A*) U2OS cells were synchronized in G2 using a thymidine block, followed by a 6-h release. Subsequently, DNA damage was induced by a 1-h doxorubicin pulse. MG132 was added after the pulse to inhibit proteasomal degradation. Cells were harvested 2 h and 6 h after DNA damage treatment for proteomics analysis. Two biological replicates were generated (*B*) proteomics platform. Following the harvest, cells were lysed and proteins were digested with trypsin. Dyglycil (di-Gly) peptides were enriched with ubiquitin remnant peptide IP. Peptides were analyzed with LC-MS, followed by MaxQuant label-free quantification. Three MS runs were performed and combined for each biological replicate. (*C*) Venn diagrams show the overlap between both biological replicates with respect to ubiquitin sites identified in the 2-h post-damage (*left*) and 6-h post-damage (*right*) time points. (*D*) Independent validation of identified ubiquitin sites. Venn diagram shows the number of ubiquitin sites identified following ubiquitin remnant peptide IP (blue) and His-ubiquitin protein IP (red). The overlap shows the number of ubiquitin sites that were identified after both enrichment methods. (*E*) Schematic representation of ubiquitin sites identified in NPM protein following di-Gly peptide IP, His-ubiquitin IP, and endogenous protein IP.

U2OS cells were grown in Dulbecco's modified Eagle's medium (DMEM) supplemented with 6% fetal bovine serum and penicillin/streptomycin. Thymidine and doxorubicin were purchased from (Sigma-Aldrich, St. Louis, Missouri) and used at 2.5 mm and 1 μm, respectively. ATM inhibitor KU55933 from (Calbiochem, San Diego, CA) and ATR inhibitor VE-821 from (Axon MedChem, Reston, VA) were used at concentration 10 μm each. MG132 was purchased from (Merck Millipore, Darmstadt, Germany) and used at 5 μm. Cycloheximide was purchased from Sigma and used between 50 and 100 μg/ml. DUB inhibitor PR-619 was from (Tebu-bio, the Netherlands) and used in the lysis buffer at the concentration of 50 μm. Cell synchronization and DNA damage application were performed as previously described (25) and outlined schematically in Fig. 1*A* and Fig. S3.

##### Antibodies

The antibody for diglycyl-remnant peptide enrichment was obtained from (Cell Signaling Technology, Danvers, MA) and used according to the standard protocol from the company. The following antibodies were used for immunofluorescence and Western blotting experiments: anti-nucleophosmin 1 and anti-nucleolin (Abcam, Cambridge, MA, 1:1,000), anti-RPL24 (Thermo Scientific, Waltham, MA, 1:1,000), anti-RPS27/27L (Thermo Scientific, 1:200), anti-RPL26 (Bethyl Laboratories, Montgomery, TX, 1:1,000), anti- RPL27a (Novus, 1:1,000), anti-RPS6 (Cell Signaling Technology, 1:500), anti-pS139-H2AX (Millipore, 1:1,000), and anti-α-tubulin (1:5,000, Sigma).

##### Nascent Protein Synthesis Analysis

G2-synchronized U2OS cells were washed with PBS and cultured in methionine-free DMEM (Invitrogen, Carlsbad, CA) for 30 min to deplete the intracellular methionine reserves. Cells were then treated with a pulse of doxorubicin for 1 h to induce double-strand breaks. After washout, cells were incubated with the methionine analog l-azidohomoalanine (AHA; Invitrogen) for 2 h; cells were treated with the translation inhibitor cycloheximide or with no AHA as negative controls. Cells were fixed in 3.7% formaldehyde, permeabilized with 0.5% Triton X-100 in PBS and blocked using 3% bovine serum albumin in PBS. Proteins containing AHA were labeled with Alexa Fluor488-alkyne using a click chemistry-based reaction (Click-iT, Life Technologies). Cells were counterstained with DAPI to stain the nuclei. The amount of nascent protein synthesis in each condition was quantified by measuring AHA fluorescence intensity per cell using a macro developed for that purpose in ImageJ.

##### Sucrose Gradients

U2OS cells were lysed in lysis buffer (20 mm Tris-HCl, pH 7.5, 10 mm MgCl_2_, 100 mm KCl, 1% NP40) supplemented with 2 mm DTT, 100 μg/ml cycloheximide, EDTA-free protease inhibitor mixture (Roche), and RiboLockRNAse inhibitor (40 U/ml, Life Technologies). Lysates were centrifuged at 1,300 *g* and the supernatant was fractionated on a linear sucrose gradient (7–47%) using a SW-41Ti rotor at 36,000 rpm for 2 h. Thirteen fractions were collected, and samples were analyzed by Western blotting using the indicated antibodies.

##### Experimental Design and Statistical Rational Preparation of Cell Lysates for Proteomics Analysis

U2OS cells were synchronized in G2 phase. Subsequently, 1-h doxorubicin pulse was applied. Upon removal of doxorubicin, cells were incubated in fresh media containing 5 μm MG132 for 2 h and 6 h and subsequently harvested for proteomics analysis.

Undamaged cells with MG132 treatment are the control for this experiment. The time scale of this experiment is presented in Fig. 1*A*. To ensure the reproducibility, two independent experiments were carried out. Each experiment contains two time points and their respective controls.

For the ATM/ATR inhibitor experiment, G2-synchronized U2OS cells were treated with 10 μm ATM- and ATR-inhibitors for half an hour before DNA damage induction. DNA damage was induced by a pulse of doxorubicin. Upon removal of doxorubicin, cells were incubated in fresh media containing 5 μm MG132 for 2 h, and cells were subsequently harvested for proteomics analysis. Doxorubicin- and MG132-treated cells without ATM and ATR inhibition are the control for this experiment. Time scale is presented in Fig. S3.

For the MG132 *versus* DMSO experiment, U2OS cells were synchronized in G2 phase and 1-h doxorubicin pulse was applied. Upon removal of doxorubicin, cells were incubated in fresh media with and without 5 μm MG132 for 2 h and subsequently harvested for proteomics analysis.

##### Protein Extraction, Proteolytic Digestion, and Peptide Purification

Harvested cells were lysed using ultrasonicator for three times 1 min at 0.6 cycle and 90% amplitude and proteins were extracted using 50 mm ammonium bicarbonate buffer containing 8 m urea, protease inhibitors, and 50 μm deubiquitinase inhibitor PR619. For each sample, 20 mg protein were reduced and alkylated using 5 mm DTT and 10 mm chloroacetamide, respectively. Subsequently, samples were digested with lys-C (1:50 w/w enzyme:protein ratio). After buffer dilution (to 2 m urea), samples were digested with trypsin (1:50 w/w enzyme:protein ratio). The peptide product was then purified using a Seppak C8 column and concentrated using a speedvac. Finally, the purified peptides were reconstituted in the immunoprecipitation buffer for further enrichment by immunoprecipiation with an antibody recognizing the diglycyl-remnant. The immunoprecipitation buffer was supplied by Cell Signaling Technology as part of the enrichment kit. Details on extraction, digestion, and peptide purification were described previously (26, 27).

##### Peptide Enrichment and MS Analysis

Following diglycyl-remnant peptide enrichment, peptides were eluted in two subsequent washes using a total of 105 μl of 0.15% TFA. Twenty-five microliter of samples were injected in triplicate into the nano-UPLC Proxeon system (Easy-nLC 1000, Thermo Scientific) coupled to an Orbitrap Elite mass spectrometer (Thermo Scientific). The injected samples were first trapped on an in-house packed trap column (ReproSil-Pur C18-AQ, 3 μm (Dr. Maisch GmbH, Ammerbuch, Germany) 2 cm × 100 μm) before being separated with 2 h gradient on an in- house made analytical column (Zorbax SB-C18, 1.8 μm (Agilent Technologies, Baltimore, MD, USA) 50 cm × 50 μm) at a constant temperature of 40 degrees. For the Orbitrap Elite a voltage of 1.7 kV was applied to the needle. The survey scan was recorded with a resolution of 60,000. The 20 most intense precursors were selected for subsequent fragmentation using HCD as the activation technique. Singly and doubly charged ions were excluded in the analysis.

##### Ubiquitin/Peptide-site Identification and Quantification, Data Analysis, and Evaluation

Raw data were processed using MaxQuant (version 1.4.0.3) (28) and the MS/MS data were queried against the human UniProt database (23,630 entries, released 2013_06). Trypsin/P was chosen as cleavage specificity allowing two missed cleavages. Carbamidomethylation (C) was set as a fix modification, while oxidation (M) and GlyGly (K) were used as variable modifications. Peptide identification was based on a search with a mass deviation of the precursor ion up to 4.5 ppm, and the allowed fragment mass deviation was set to 20 ppm for Fourier transformation mass spectrometry (FTMS) and 0.5 Da for ion trap mass spectrometry (ITMS). Data filtering was carried out using the following parameters: peptide and protein false discovery rate were set to 1%, minimum peptide length was set to 6, and Andromeda minimum score was set to 40 (29). MaxQuant label-free quantification was used to quantify the ubiquitin- peptide/site, with peak area as the output. To analyze the data, peak area of treated samples was compared with their respective controls such as with and without damage 2 h; with and without damage 6 h; with and without ATM-/ATR- inhibitor; with and without MG132. Log scale 2 was used to present the ratio proportionally. Data imputation was done using the lowest peak area quantified in the same run. Quantified sites were evaluated with Perseus (version 1.4.0.8) (30). Significant B is an outlier test provided by Perseus to calculate the significance of a ratio based on a ratio population (from the total data) binned by log intensity. Only ubiquitin sites that obtained a *p* < 0.01 value across replicates were considered as changed sites. Ingenuity analysis was used to further identify common changes in the system in response to the treatment.

## RESULTS

### 

#### 

##### Profiling of Protein Ubiquitination in Response to DNA Double-strand Breaks

To analyze global changes of protein ubiquitination in response to DNA damage, G2- synchronized U2OS cells were either left untreated or treated with a pulse of doxorubicin to induce double-strand breaks. After doxorubicin washout, cells were cultured in the presence of the proteasome inhibitor MG132 to prevent the degradation of ubiquitinated proteins, and harvested 2 h and 6 h later to profile protein ubiquitination (Fig. 1*A*). Proteins were extracted, digested with trypsin, and the ubiquitinated peptides were enriched using a diglycyl remnant peptide antibody (31). Subsequently, peptides were analyzed by LC-MS and quantified using MaxQuant Label-Free Quantification (32) (Fig. 1*B*). Two independent biological replicates were performed for each experiment.

As expected, treatment with MG132 markedly increased the abundance of diglycine peptides compared with the DMSO-treated control (Fig. S1*A*) and allowed us to detect ubiquitinated proteins that otherwise would have been degraded by the proteasome in the absence of MG132. Importantly, upon treatment with doxorubicin and MG132, the overall levels of ubiquitin conjugates did not change (Fig. S1*B*), and the types of ubiquitin linkages were not significantly altered (Fig. S1*C*).

In total, we could identify more than 10,000 ubiquitin sites at each time point, with an overlap of ∼90% of identified and quantified sites between each biological replicate (Fig. 1*C*). The overlap in sites quantified at each time point was also high (87%, Fig. S1*D*). This leads to a total of more than 11,000 unique ubiquitin sites (Table S1). To validate some of the sites identified in our large-scale analysis, we expressed a His-tagged variant of ubiquitin in U2OS cells. After lysis, proteins modified with His-tagged ubiquitin were pulled-down using nickel charged beads, digested, and the ubiquitinated peptides were immunoprecipitated using the ubiquitin remnant antibody. We identified a total of 836 unique ubiquitinated peptides, 469 of which overlapped with the peptides we identified in our large-scale proteomics (Table S2 and Fig. 1*D*). Thus, close to 60% of the ubiquitin sites identified by His-ubiquitin tagging were also identified in the direct ubiquitin remnant isolation (Fig. 1*D*). While this provides some validation to our large-scale ubiquitin analysis approach, it also shows that a substantial amount of protein ubiquitination was missed in the direct ubiquitin remnant pull-downs as compared with the His-ubiquitin pull-downs and vice versa.

As an example of our validation, the highly ubiquitinated NPM1 protein is shown (Fig. 1*E*). Our initial large-scale proteomics identified six ubiquitin-sites (K32, K239, K248, K250, K257, and K273) in NPM1. Using His-ubiquitin protein Immunoprecipitation (IP) as well as endogenous protein IP, we were able to independently validate the ubiquitination on K239, K248, K257, and K273 in NPM1. Combined, our validation experiments indicate the robustness of our large-scale ubiquitin analysis approach and thereby strengthen our findings.

##### Changes in Protein Ubiquitination After DNA Damage

**Fig. 2.**
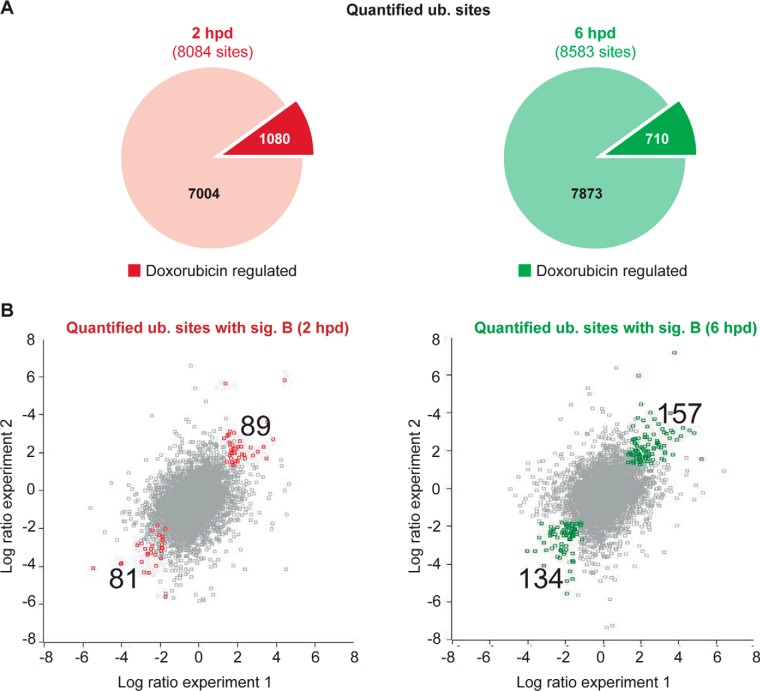
**Ubiquitome data evaluation.** (*A*) Pie charts showing the proportion of doxorubicin-regulated ubiquitin sites over the total number of quantified ubiquitin sites at each time point. The ubiquitin sites whose abundance increased or decreased at least twofold following doxorubicin treatment were considered doxorubicin-regulated. (*B*) Scatter plots represent the correlation of the log2 ratios of doxorubicin-regulated ubiquitin sites between the two independent experiments. Colored dots indicate sites that are significantly regulated in both experiments (*p* < 0.01, significant B test).

Using label-free quantification of diglycyl remnant peptides, we determined changes in abundance of ubiquitinated peptides between the damaged and control samples. To obtain an overview of all of the changes in protein ubiquitination, we subjected all of the unique sites that were identified at each single time point to a significant B test considering both intensity and ratio (*p* < 0.01). This analysis provided us with a list of 1,732 peptides from the 2 h time points (Table S3) and 2,319 peptides from the 6 h time points (Table S4) for which the intensity changed at least twofold between the doxorubicin-treated and the control samples. We find that ∼10% of all the identified ubiquitin sites were significantly changed in each individual run (Fig. 2*A*). Gratifyingly, and in line with the important role of ubiquitination in DNA replication, recombination, and repair, we identified many proteins that were previously shown to be regulated by ubiquitination/deubiquitination in response to DNA damage, such as Brca1, FANCD2, FANCI, BLM, PCNA, Cdc25A, DDB2, and Histone H2A.X (Table S1), supporting the quality of our data (33–37).

To identify the most reliable changes in protein ubiquitination that take place in response to DNA damage, we subsequently extracted only those peptides that passed the significant B test in both independent experiments for each single time point. This resulted in a list of 461 sites that were reproducibly regulated in both independent experiments (Tables S5 and S6). We identified 170 lysines 2 h after damage (81 decreasing and 89 increasing) and 6 h after damage this number increased to 291 lysines (134 decreasing and 157 increasing) (Fig. 2*B*, Table S5 and S6). The relative overlap in significantly regulated sites when comparing the 2 h and 6 h time points was relatively low (5%), implying that many of the ubiquitination events are transient and reversible. It was reassuring to find that the list of most reliable changes in protein ubiquitin contained a large variety of DNA-damage-related proteins (DNA- PK, Nek2, BRAT1, DDIT4, nucleophosmin, DNA polymerase delta epsilon, HERC2, CHD4, PCNA, BRAP, RAP80, BRCA1, BLM) (Tables S5 and S6). But more strikingly, we noticed a very high proportion of ribosomal and nucleolar proteins to be enriched in the list of proteins whose ubiquitination increased upon doxorubicin treatment (Tables S5 and S6).

**Fig. 3.**
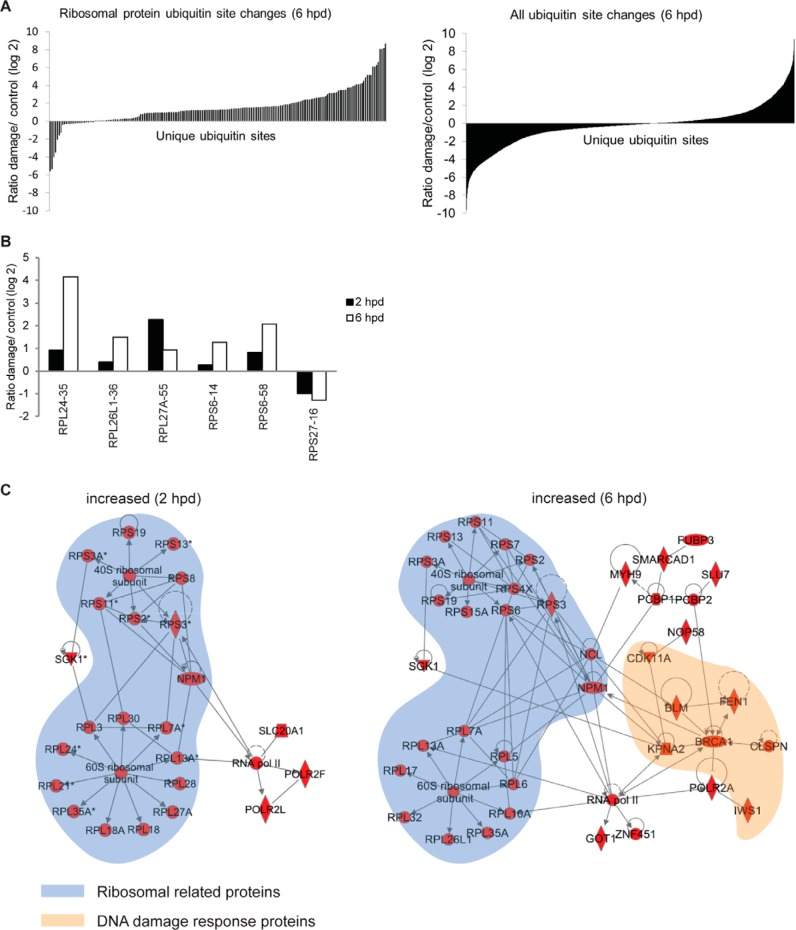
**Ribosomal protein ubiquitome data evaluation.** (*A*) Plots showing the log2(damage/control) ratios of quantified ubiquitin sites on ribosomal proteins (*left panel*) *versus* all identified proteins (*right panel*), at the 6 h post-damage time point, sorted according to their ubiquitination/deubiquitination status. (*B*) Log2(damage/control) ratios for ubiquitinated sites on the ribosomal proteins RPL24, RPL26L1, RPL27A, RPS6, and RPS27, both at 2 h and 6 h post-damage time points. The ubiquitinated site is indicated after the name of the protein. (*C*) Interaction networks of proteins with ubiquitination sites showing a significant change 2 h (*left*) and 6 h (*right*) after the DNA damage pulse. The highest scoring network (according to Ingenuity Pathway Analysis) for each time point is plotted. Arrows indicate an interaction, and lines without arrowheads indicate binding. Ribosomal and nucleolar proteins are highlighted in blue and DNA damage response proteins, which are enriched in the 6 h time point, are highlighted in orange.

To confirm the overrepresentation of ribosomal proteins in the ubiquitinated protein list, we sorted all identified ubiquitin sites based on their ratio (Fig. 3*A*). Compared with the sorted list of all ubiquitin-site changes, where ubiquitination/deubiquitination is more or less equal (Fig. 3*A*, *right panel*), we observed prominent ubiquitination of ribosomal and nucleolar proteins after the DNA damage pulse (Fig. 3*A*, *left panel*; Tables S5 and S6). In addition, protein deubiquitination is also observed at a few sites in the ribosomal proteins, indicating that ubiquitination of ribosomal proteins upon damage is not necessarily uniformly regulated. This is further supported by the differences we observe in the relative ratios of six ubiquitin sites of different ribosomal proteins at both 2 h and 6 h after the DNA damage pulse (Fig. 3*B*). Next, we used Ingenuity Pathway Analysis to confirm that indeed, the ribosomal pathway was identified as a target of protein ubiquitination after DNA damage. The highest scoring networks identified by Ingenuity Pathway Analysis for the 2 h and 6 h time points, respectively, are depicted in Fig. 3*C*, and strikingly, both contain a high proportion of ribosomal proteins. To confirm that activation of the DNA damage checkpoint results in increased ubiquitination of ribosomal proteins, we performed the same ubiquitin profiling described above in the presence of ATM and ATR inhibitors (Fig. S2*A*), and the changes in protein ubiquitination were analyzed. Indeed, we found that the ubiquitination of ribosomal and nucleolar proteins was mostly dependent on ATM and ATR, since inhibition of these kinases largely prevented the changes in protein ubiquitination after DNA damage (Fig. S2*B*). These data suggest that activation of the DNA damage checkpoint results in a substantial, ATM/ATR-dependent change in ubiquitination of ribosomal and nucleolar proteins.

We next compared our results with two recently published large-scale ubiquitin studies (8, 38). We used the 2 h time point from our data set because this most closely reflected the time points used to obtain the data sets in DTT- and ionizing-radiation-treated cells to induce endoplasmic reticulum (ER)- and DNA-damage stress, respectively (Fig. S4 and Table S7). While the overlap in the overall data sets was low, DTT-induced ER stress also triggered widespread site-specific ubiquitination of ribosomal proteins. Further analysis showed that a number of ribosomal proteins were ubiquitinated in response to ER- as well as after DNA-damage-induced stress (Fig. S4 and Table S7). For example, our analysis identified five ubiquitin sites on RPS3, all of which were also up-regulated in response to ER stress. In addition, while no common ubiquitin sites of RPS2 and RPS20 were found when comparing ER- and DNA-damage-induced stress, both proteins are ubiquitinated in response to DNA damage and ER stress. Thus, despite a very limited overall similarity in the outcome of the different screens, ubiquitination of ribosomal proteins appears to be a common response of cells to different forms of stress.

##### DNA Damage Results in a Decrease in General Protein Translation

**Fig. 4.**
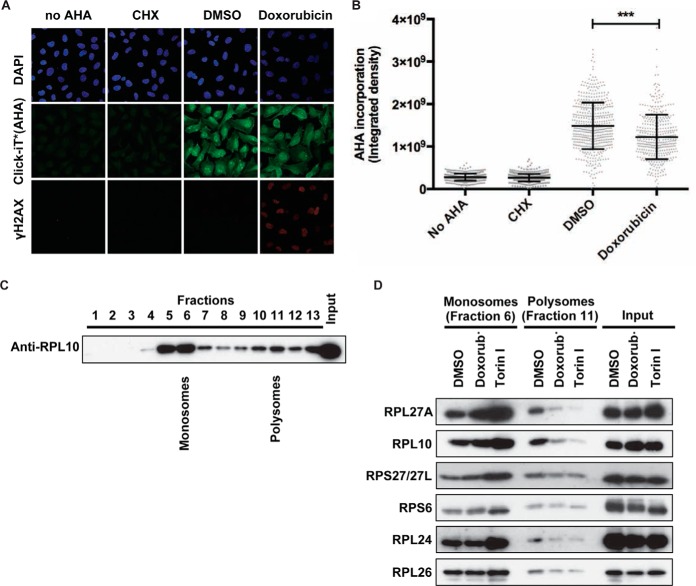
**DNA damage affects ribosomal function.** (*A*) U2OS cells were synchronized in G2 as described for Fig. 1(*A*) and treated with doxorubicin for 1 h in methionine-depleted media. After wash out, cells were incubated with l- azidohomoalanine (AHA) for 2 h; cells with no AHA or treated with cycloheximide were used as negative controls. Cells were fixed and incubated with Alexa Fluor 488 alkyne to label AHA incorporation into nascent proteins; cells were counterstained with DAPI to show the nuclei. γH2AX staining is used as a marker for DNA break formation. Panels show representative confocal images from each condition. (*B*) Scatter plot of individual AHA levels in no AHA, cycloheximide-, DMSO-, and doxorubicin-treated samples from one out of the six experiments. The amount of nascent protein synthesis in each condition was quantified by measuring AHA fluorescence intensity per cell using a macro developed for this purpose. Each bar represents mean ± S.D. from each condition. Statistical significance was determined using nonparametric Kruskal-Wallis test (***p < 0.0001). (*C*) Lysates from DMSO-treated U2OS cells were fractionated in sucrose density gradients to isolate the monosomes and polysomes. Thirteen fractions and the input were separated by SDS-PAGE and immunoblotted with anti-RPL10 antibody. Fractions 6 and 11 were assigned as representative monosome- and polysome-enriched fractions. (*D*) Lysates from cells treated with DMSO, doxorubicin, and Torin I were fractionated in sucrose density gradients to isolate the monosomes and polysomes. Fractions 6 and 11 from each sample were separated by SDS-PAGE and immunoblotted with indicated antibodies.

The function of nucleoli and ribosomes in protein synthesis is well established (39). Since we find that a large number of nucleolar and ribosomal proteins are ubiquitinated following DNA damage, this sparked our interest to study the effect of DNA damage on protein synthesis. Therefore, we analyzed changes in global nascent protein synthesis following DNA damage. To this end, AHA, an analog of methionine amino acid, was added to the cell culture following a doxorubicin pulse, and incorporation of AHA into newly synthesized proteins was measured by Click-iT reaction (40, 41). Significantly less AHA incorporation was observed in doxorubicin-treated cells compared with DMSO-treated cells, indicating a decrease in nascent protein synthesis following DNA damage (Fig. 4*A* and 4*B*). This decrease in nascent protein synthesis was also observed when doxorubicin was combined with the proteasome inhibitor MG132 (Fig. S4*A* and S4*B*), indicating that the effects we observe on ubiquitination of ribosomal proteins and protein synthesis are primarily caused by the DNA damaging insult.

Next, we aimed to test whether the decrease in global protein synthesis was accompanied by a change in ribosome activity. Protein synthesis involves translation of mRNA strands by ribosomes. During active protein synthesis, several ribosomes are attached to a single mRNA strand simultaneously, forming so-called polysomes. When not translationally active, polysomes dissociate again into monosomes. Both monosomes and polysomes can be separated on sucrose gradients and identified by immunoblotting with antibodies against ribosomal proteins (Fig. 4*C*). As a control, we inhibited mRNA translation using an mTOR inhibitor (Torin I), which results in a clear loss of polysomes (Fig. 4*D*). Similarly, a reduction of polysomes in the doxorubicin-treated cells is observed (Fig. 4*D*), indicating a decrease in ribosome activity in response to DNA damage. Together, these results indicate that protein translation is affected by DNA damage stress.

##### DNA Damage Affects the Subcellular Localization of Ribosomal Proteins

**Fig. 5.**
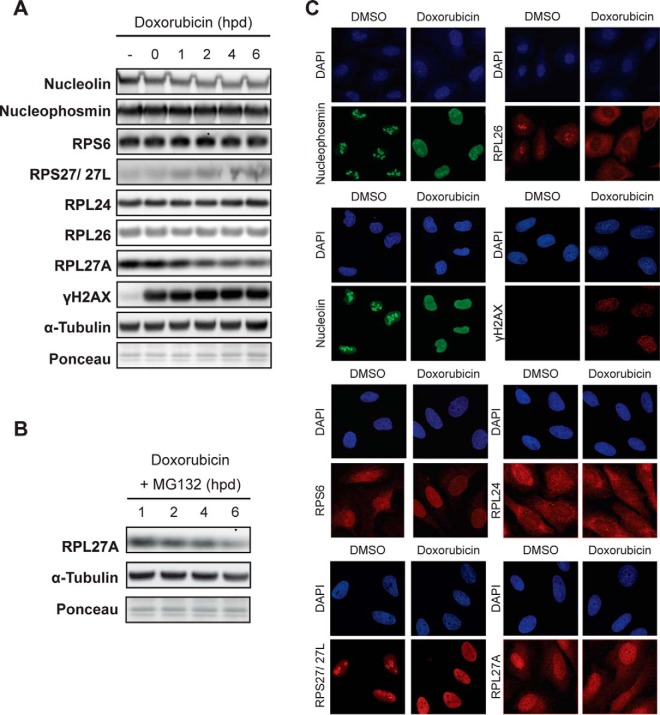
**Expression level and localization of nucleolar and ribosomal proteins in response to DNA damage.** (*A*) Expression level of ribosomal and nucleolar proteins after DNA damage. U2OS cells were synchronized in G2 and treated with a pulse of doxorubicin for 1 h. Cells were harvested at the indicated hours post-damage (hpd), and the expression of several proteins was analyzed with the indicated antibodies. Tubulin and ponceau S were used as loading controls. Both in (*A*) and (*C*), γH2AX was used as a marker for DNA damage. (*B*) Same as in (*A*) but with the inclusion of MG132 after the damaging insult. (*C*) Cellular localization of ribosomal and nucleolar proteins after DNA damage. U2OS cells were synchronized in G2, fixed 2 h after doxorubicin pulse and stained with the indicated antibodies using immunofluorescence. Cells were counterstained with DAPI to show the nuclei.

Depending on the type of linkage, ubiquitination can promote protein degradation or alter protein behavior. Particularly in the DNA damage response, protein ubiquitination is known to play an important role in checkpoint signaling and repair by controlling protein function. To study the consequences of ubiquitination of ribosomal proteins, we first studied if DNA damage induces degradation of ribosomal proteins. We harvested cells at different time points after doxorubicin treatment and analyzed expression levels of several ribosomal proteins by Western blotting using commercially available antibodies. Expression of nucleolin, nucleophosmin, RPS6, RPL24, and RPL26 remained constant throughout the experiment (Fig. 5*A*). Expression of RPS27/27L seemed to increase during the course of the experiment, whereas expression of RPL27A was reduced over time (Fig. 5*A*). The reduction in RPL27A expression was not reverted by addition of MG132, indicating that this reduction is not due to enhanced proteasomal degradation (Fig. 5*B*). Thus, we can conclude that, at least for the ribosomal proteins analyzed here, DNA damage does not seem to induce increased proteasomal degradation. This does not exclude the possibility that ubiquitination can induce degradation of some of the other ribosomal proteins that we find to be ubiquitinated after DNA damage, but at least this shows that DNA damage does not decrease stability of ribosomal proteins in general.

Nucleolar stress produced by DNA damage has been shown to promote the translocation of nucleolin and nucleophosmin from the nucleolus to the nucleoplasm, and therefore we analyzed if ubiquitination affects subcellular localization of other ribosomal proteins in a similar manner. As expected, we find that both nucleolin and nucleophosmin are dispersed from the nucleolus to the nucleoplasm in response to treatment with doxorubicin (Fig. 5*C*). In addition to nucleolin and nucloephosmin, both RPL26 and RPS27/27L were dispersed from the nucleoli after a pulse of doxorubicin (Fig. 5*C*), but while RPS27/27L accumulated in the nucleoplasm, RPS26 was found in the the cytoplasm (Fig. 5*C*). No clear differences in subcellular localization were observed for RPS6, RPL24 and RPL27A, but none of these latter ribosomal proteins accumulated in the nucleoli in the untreated cells either (Fig. 5*C*). These data indicate that the enhanced ubiquitination of ribosomal proteins that we observe after doxorubicin coincides with nucleolar stress and disruption of the nucleoli. Based on our data, we cannot discriminate if ubiquitination is involved in the onset of nucleolar disruption, or if it occurs as a consequence of this disruption. However, the fact that we find a clear increase in ubiquitination of ribosomal proteins as early as 2 h after the damaging insult could be compatible with a role in the disruption of the nucleolus itself.

## DISCUSSION

In the study described here, we have profiled global changes of protein ubiquitination in response to doxorubicin-induced DNA damage. In a previous study, ionizing radiation was used to induce DNA damage, and protein ubiquitination during the early stages of DNA damage signaling was analyzed (8). In turn, our study focuses on the analysis of ubiquitination not only at early time points but also at relatively late time points after doxorubicin-induced DNA damage. The different experimental approaches used in each study highlights the complementary of our work, and explains the limited overlap between them (Fig. S3).

Similar to other studies on protein ubiquitination, we made use of the proteasomal inhibitor MG132 to increase the chance to retrieve ubiquitination sites before their degradation (8). This has the potential caveat that addition of MG132 might also lead to depletion of the free ubiquitin pool and thereby restrict nondegradative ubiquitination. As such, it is important to strike a balance between the inhibition of ubiquitin-dependent protein degradation and the availability of free ubiquitin. This can be achieved by applying relatively low MG132 concentration in a short time period, similar to the conditions used in the present study and other previous works (34, 36, 42). Importantly, we were able to show that MG132 itself does not grossly alter DNA-damage-induced ubiquitination (Fig. S1). Also, the large amount of ubiquitination sites identified in each experiment (∼9,000), suggests that the free ubiquitin pool was not overly compromised. Moreover, the detection of well-known monoubiquitinated sites in proteins such as FANCD2 and FANCI (K561 and K523, respectively, (see Tables S3 and S4) suggests that our MG132 treatment did not severely compromise the identification of nondegradative ubiquitination sites. On the other hand, it is important to note that the ubiquitin sites were enriched and identified based on the diglycil remnant on the peptides following a tryptic digestion. Consequently, we cannot exclude the presence of some other ubiquitin-like molecules containing C-terminal diglycil motifs in the data set (34, 43). These include neddylated proteins, whose role in DNA damage response has previously been reported (43). Also, without an analysis at the proteome level we cannot deconvolute the contribution of change in protein expression on the change in level of ubiquitination observed.

We find that at 6 h post-doxorubicin treatment, proteins with a function in the DNA replication, recombination, and repair are substantially ubiquitinated. Network analysis also showed that a large group of ribosomal and nucleolar proteins are ubiquitinated in response to DNA damage. Since the main function of the ribosome is to synthesize proteins, we hypothesized that DNA-damage-induced signaling could suppress ongoing protein translation, as previously reported after other types of DNA damage such as γ-irradiation or UV (44, 45) The resulting inability to generate new proteins could help preventing further progression through the cell cycle, allowing more time to repair the damage (46). Indeed we find that DNA damage results in a rapid inhibition of protein synthesis.

But how does DNA damage control protein synthesis? DNA damage can affect initiation of protein translation through the mTOR signaling pathway (47–49), but additional mechanisms that control protein synthesis in response to DNA damage might very well exist. Recently, Higgins *et al.* proposed site-specific regulatory ubiquitination of 40S ribosomal proteins as a novel mechanism to inhibit protein translation in response to cellular stress, in particular following ionizing radiation or DTT treatment (8, 38). Similar to those studies, we also find extensive ubiquitination of ribosomal proteins following doxorubicin treatment, several of which identical to proteins identified in these earlier studies (RPS2, RPS3, RPS20) (Fig. S3 and Table S7). Moreover, we have identified many additional sites on ribosomal proteins that are ubiquitinated in response to doxorubicin, and that could also affect ribosome activity. Alternatively, DNA damage could affect ribosome biogenesis and in this way inhibit protein translation in a more general fashion. Indeed, we observe a clear nucleolar stress response after doxorubicin treatment, as both nucleolin and nucleophosmin disperse from the nucleolus. Translocation of nucleophosmin during nucleolar stress was recently shown to require S-glutathionylation, which occurs within minutes and can promote the activation of p53 (50). Our measurements of protein ubiquitination were performed as early as 2 h after the damage, at a time point when the nucleolar dispersement is well underway. Thus, further experiments are required to resolve if the ubiquitination plays a role in the translocation of ribosomal proteins from the nucleolus or if it is a mere consequence of the dispersal.

It is tempting to speculate that ubiquitination of ribosomal proteins is involved in the inhibition of protein translation that we observe in response to doxorubicin treatment. Given the large number of ubiquitination events, it will be challenging to provide direct evidence for this, since each single event could potentially contribute to it. Nonetheless, closer examination of a selected number ubiquitination sites could prove very informative. RPS6, for example, is a component of the 40S subunit and localizes at the interface between two ribosomal subunits. It interacts with mRNA, tRNA and initiation factors (22, 49), indicating that it sits at an important interface during protein translation. Therefore, an in-depth analysis of RPS6 ubiquitination, combined with their effects on protein translation is likely to generate interesting insights. In addition, ribosomal proteins can also be highly selective in controlling protein translation. For example, RPL26 can specifically control p53 translation by interacting with the p53 mRNA (18). At the same time, p53 has been reported to induce the expression of RPS27L after the treatment with the DNA damaging agent etoposide (51), a similar increase to what we observe in response to doxorubicin (Fig. 5*A*).

We find extensive ubiquitination of ribosomal proteins at 2 h and 6 h after the induction of DNA damage. Interestingly, Elia and co-workers reported no significant change in ubiquitination of ribosomal proteins at earlier time points after damage (8). Thus, it is possible that ribosomal protein ubiquitination is part of the intermediate-to-late DNA damage response. It would therefore be interesting to study the role of ribosomal proteins during recovery from a DNA-damage-induced arrest. In this respect, it is interesting to note that we find that depletion of several ribosomal proteins results in a substantial decrease in recovery from a G2 arrest (data not shown). Similarly, depletion of RPS27L resulted in a deficiency in DNA damage checkpoints, leading to a shift of DNA-damage-induced p53 response from cell cycle arrest to apoptosis (52). How ribosomal proteins or their ubiquitination affects recovery is not clear. Recently, an E3 ligase complex with a role in nonfunctional rRNA decay has been identified and its associated protein Mms1p, identified previously as factor involved in DNA repair (53). Several studies have linked ribosomal proteins to the activation of p53, controlling its abundance either by binding to p53 mRNA or by binding to its ubiquitin ligase MDM2 (17, 54–56). Given the crucial role for p53 in the control of cell cycle re-entry in G2 (57, 58) these extra-ribosomal functions could prove important.

In summary, our proteomics study provides a useful data set of protein ubiquitination events that occur in response to DNA damage. Our set further expands previously published data sets and provides the first global analysis of protein ubiquitination at early and late time points after treatment with doxorubicin. In addition, our study highlights the extensive effects of DNA damage on ubiquitination of ribosomal proteins, as well as its effects on protein synthesis. While we would like to unravel the molecular details that could link these events, the large number of ubiquitination events that we and others find represents a huge challenge. Nonetheless, the fact that many subunits of the ribosome are affected during a stress response strongly implies that tight control of ribosomal function is crucial for the cellular response to stress.

## Data Availability

All raw files and annotated spectra from these experiments are available on PRIDE (https://www.ebi.ac.uk/pride/archive; Project ID PXD004445).

## Supplementary Material

Fig. S3
