# Supplementary material for: Doxorubicin-induced DNA Damage Causes Extensive Ubiquitination of Ribosomal Proteins Associated with a Decrease in Protein Translation*
Source: Mol Cell Proteomics. 2018 Feb 8;17(12):2297–308. doi: 10.1074/mcp.RA118.000652 (PMC6283304; doi:10.1074/mcp.RA118.000652)
Supplement: Fig. S3 [file RA118.000652_index.html]

Supplement to Doxorubicin-induced DNA damage causes extensive ubiquitination of ribosomal proteins associated with a decrease in protein translation | Molecular & Cellular Proteomics

## Supplemental Data

- Supplemental tables S1-S7 - Supplemental Tables Table S1. Identified ubiquitin sites using the Gly-remnant IP. 11172 ubiquitin sites were identified from 33 LC-MS/MS runs. In this study ubiquitin-enriched peptide samples (doxorubicin-treated U2OS cells and its respective controls) were analyzed with Orbitrap LC-MS, followed by MaxQuant label-free quantification. Log scale 2 was used to present the ratio proportionally. For data filtering protein and peptide FDR were set to 1%. Table S2. Identified ubiquitin sites using the His-Ubiquitin IP. 836 ubiquitin sites were identified from 2 LC-MS/MS runs (doxorubicin treated U2OS cells and its respective controls). The 469 ubiquitin sites that were also identified in the diglycyl-remnant enrichment (see Table S1) are highlighted in red. Table S3. Ubiquitin sites with ratios exhibiting significance from the 2 hr time-points. Quantified sites were evaluated with Perseus. Ubiquitin sites that passed the significant B-test were considered as changed sites and marked as "+" under sig.B. column. Table S4. Ubiquitin sites with ratios exhibiting significance from the 6 hr time-points. Quantified sites were evaluated with Perseus. Ubiquitin sites that passed the significant B-test were considered as changed sites and marked as "+" under sig.B. column. Table S5. Significant changes in ubiquitination at 2h post damage (n=2). Only ubiquitin sites that significantly changed in two independent experiments are listed. Ubiquitin sites that passed the significant B-test were considered as changed sites and marked as "+" under sig.B. column. Table S6. Significant changes in ubiquitination at 6h post damage (n=2). Only ubiquitin sites that significantly changed in two independent experiments are listed. Ubiquitin sites that passed the significant B-test were considered as changed sites and marked as "+" under sig.B. column. Table S7. Differentially ubiquitinated proteins after doxorubicin-treatment with ratios exhibiting significance that overlap with differentially ubiquitinated proteins identified in other recent studies (8,38). Overlapping and Non-overlapping protein ubiquitination induced by doxorubicin treatment (this study), DNA damage stress (8) and ER-stress (38) are listed.
- Figure S1 - Figure S1. Evaluating the effect of MG132 on DNA damage-induced ubiquitination. (A) Number of quantified diglycil peptides in DMSO- and MG132-treated DNA-damaged U2OS cells. (B) Expression of ubiquitin conjugates following DNA damage. U2OS cells were synchronized in G2 and treated with a pulse of doxorubicin for one hour. Following doxorubicin pulse, new media containing MG132 was applied. Cells were harvested at the indicated hours post damage (hpd), and the expression of all ubiquitinated proteins was analyzed with the anti-FK2 antibody. &#x03B1;-tubulin was used as a loading control. (C) Plot showing the intensity (in log10 scale) of total ubiquitin sites with different types of linkages (K6, K11, K27, K29, K33, K48, K63) in each timepoint and condition. (D) Venn diagram indicating the overlap of quantified ubiquitin sites in each timepoint.
- Figure S2 - Figure S2. Effect of ATM and ATR kinase inhibition on DNA damage-related ubiquitination of ribosomal and nucleolar proteins. (A) Experimental setting of ATM- and ATR- inhibition experiment. U2OS cells were synchronized in G2 phase as described for Fig. 1A. ATM- and ATR- inhibitors were applied half an hour before DNA damage induction. DNA damage was induced by a pulse of doxorubicin and proteosomal degradation was inhibited by MG132 as described for Fig. 1A. Cells were harvested two hours after DNA damage. (B) Heat map presenting the effect of ATM and ATR kinase inhibition on ribosomal protein ubiquitylation. The number following the gene symbol indicates the ubiquitin site. Red is two-fold or more decreased; yellow is unchanged; green is two-fold or more increased.
- Figure S3 - Figure S3. Overlap with other studies. Venn diagram comparing the proteins found to be ubiquitinated in our study two hours after doxorubicin pulse (red circle), with two recent similar studies where cells were treated with ionizing radiation (green circle) (8) or ER stress (blue circle) (38). Data from 2 independent experiments are combined.
- Figure S4 - Figure S4. DNA damage affects ribosomal function. (A) U2OS cells were synchronized in G2 as described for Fig. 1A, and treated with doxorubicin for 1 hour in methionine-depleted media. After washout, cells were incubated with L-azidohomoalanine (AHA) for 2 hours in the presence of MG132; cells with no AHA or treated with cycloheximide (CHX) were used as negative controls. Cells were fixed and incubated with Alexa Fluor 488 alkyne to label AHA incorporation into nascent proteins; cells were counterstained with DAPI to show the nuclei. &#x0263;H2AX staining is used as a marker for DNA break formation. Panels show representative confocal images from each condition. (B) Scatter-plot of individual AHA levels in No AHA, CHX-, MG132- and MG132+doxorubicin- treated samples from a representative experiment. The amount of nascent protein synthesis in each condition was quantified by measuring AHA fluorescence intensity per cell using a macro developed for this purpose. Each bar represents mean &#x00B1; SD from each condition.
- Legends supplementary tables and figures - Legends to supplementary tables and figures
