# Supplementary figures and images for: Doxorubicin-induced DNA Damage Causes Extensive Ubiquitination of Ribosomal Proteins Associated with a Decrease in Protein Translation*
Source: Mol Cell Proteomics. 2018 Feb 8;17(12):2297–308. doi: 10.1074/mcp.RA118.000652 (PMC6283304; doi:10.1074/mcp.RA118.000652)

Figure S1

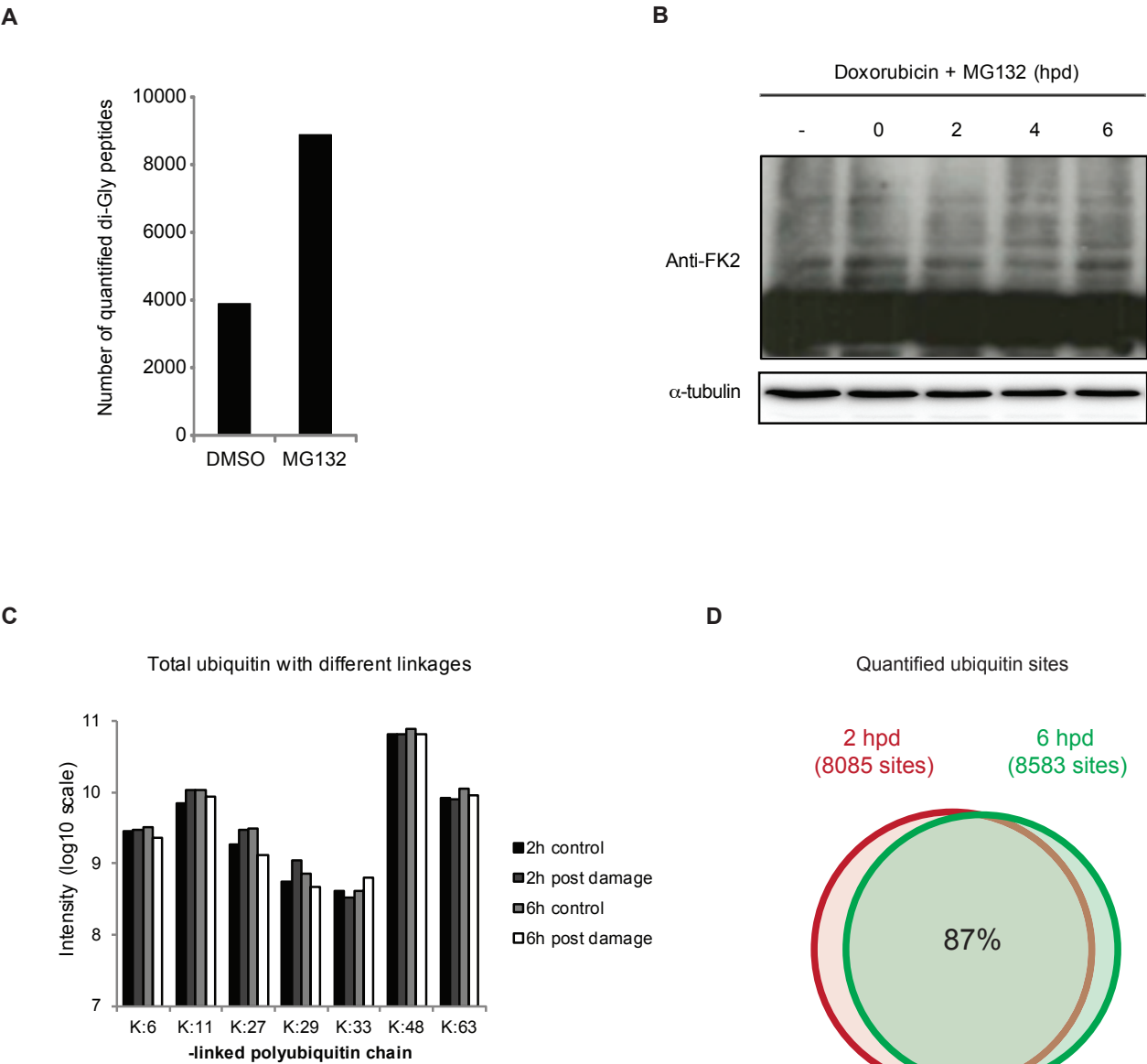

Supplement: Fig. S3 [file 135740_0_supp_70435_p3qzd5.pdf]

Figure S3

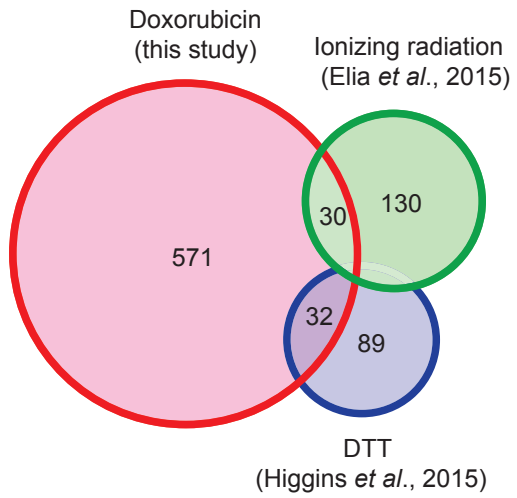

Supplement: Fig. S3 [file 135740_0_supp_70437_p3q6d5.pdf]

Figure S4

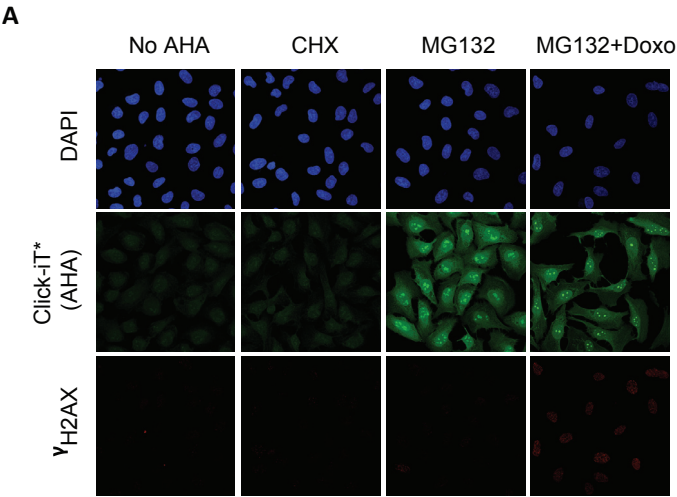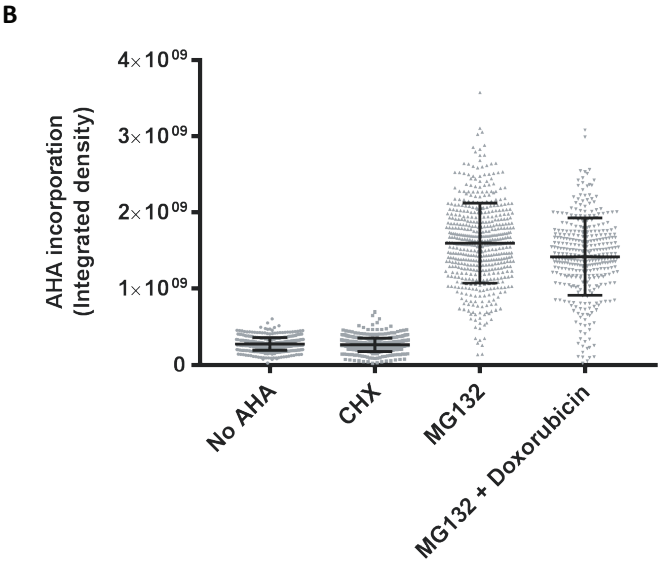

Supplement: Fig. S3 [file 135740_0_supp_70438_p3qjd5.pdf]
