# Supplementary material for: Doxorubicin-induced DNA Damage Causes Extensive Ubiquitination of Ribosomal Proteins Associated with a Decrease in Protein Translation*
Source: Mol Cell Proteomics. 2018 Feb 8;17(12):2297–308. doi: 10.1074/mcp.RA118.000652 (PMC6283304; doi:10.1074/mcp.RA118.000652)
Supplement: Fig. S3 [file 135740_0_supp_70436_p3qzd5.pdf]

**Figure S2**

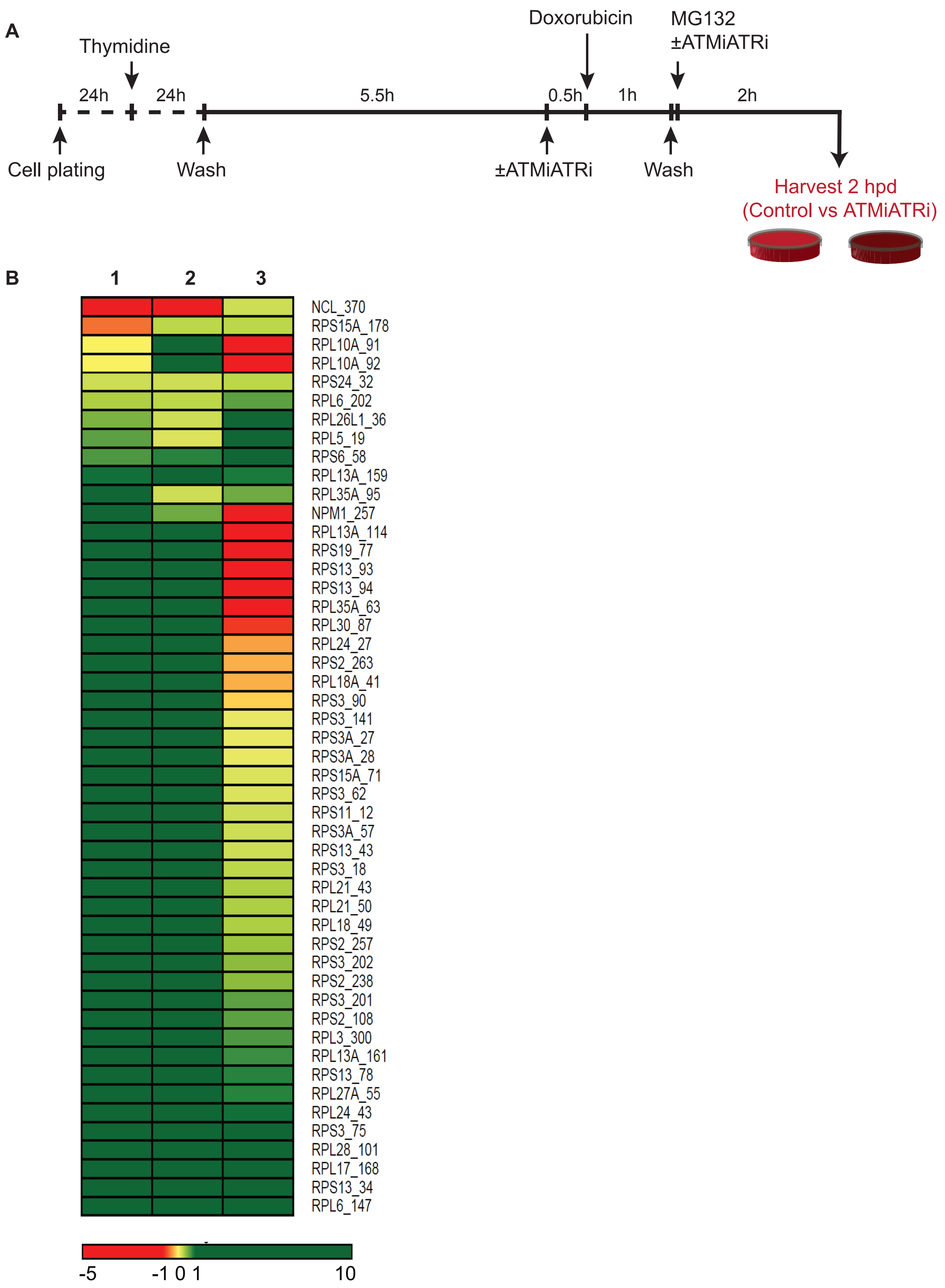


1: Log ratio w/ wo- damage 2h + MG132 (Exp1)
2: Log ratio w/ wo- damage 2h + MG132 (Exp2)
3: Log ratio damage 2h + MG132;
w/wo - ATM &ATR inhibitor
